# “Monitoring inflammatory, immune system mediators, and mitochondrial changes related to brain metabolism during space flight”

**DOI:** 10.3389/fimmu.2024.1422864

**Published:** 2024-10-01

**Authors:** Darcy Tocci, Tomas Ducai, C. A. Barry Stoute, Gabrielle Hopkins, Mohammad G. Sabbir, Afshin Beheshti, Benedict C. Albensi

**Affiliations:** ^1^ Barry & Judy Silverman College of Pharmacy, Nova Southeastern University, Fort Lauderdale, FL, United States; ^2^ Center for Molecular Biology, University of Vienna, Vienna, Austria; ^3^ Consultant, Gatineau, QC, Canada; ^4^ College of Psychology, Nova Southeastern University, Fort Lauderdale, FL, United States; ^5^ McGowan Institute for Regenerative Medicine - Center for Space Biomedicine, Department of Surgery, University of Pittsburgh, Pittsburgh, PA, United States; ^6^ Broad Institute, Cambridge, MA, United States; ^7^ Max Rady College of Medicine, University of Manitoba, Winnipeg, MB, Canada; ^8^ Division of Neurodegenerative Disorders, St. Boniface Hospital Research, Winnipeg, MB, Canada

**Keywords:** space flight, space medicine, countermeasures, brain, aging, cognition, memory, CNS

## Abstract

The possibility of impaired cognitive function during deep space flight missions or while living on a Martian colony is a critical point of concern and pleads for further research. In addition, a fundamental gap exists both in our understanding and application of countermeasures for the consequences of long duration space travel and/or living in an extreme environment such as on the Moon or Mars. Previous studies, while heavily analyzing pre- and post-flight conditions, mostly fail to appreciate the cognitive stressors associated with space radiation, microgravity, confinement, hostile or closed environments, and the long distances from earth. A specific understanding of factors that affect cognition as well as structural and/or physiological changes in the brains of those on a space mission in addition to new countermeasures should result in improved health of our astronauts and reduce risks. At the core of cognitive changes are mechanisms we typically associate with aging, such as inflammatory responses, changes in brain metabolism, depression, and memory impairments. In fact, space flight appears to accelerate aging. In this review, we will discuss the importance of monitoring inflammatory and immune system mediators such as nuclear factor kappa B (NF-κB), and mitochondrial changes related to brain metabolism. We conclude with our recommended countermeasures that include pharmacological, metabolic, and nutritional considerations for the risks on cognition during space missions.

## Introduction

In the summer of 1969 the world watched with bated breath as the first humans to land on the moon made their debut (1–3). Forge ahead to present day and we see the advancement of space exploration unfolding at rates we had never imagined and with new challenges that we have only recently imagined (4–8). With the increasing likelihood that future exploratory missions will be vastly greater, both in duration and distance, comes the impending need to equip our crew members with the latest advancements in cognitive health (9–13) from neuroscience research. For example, plans to send humans to Mars are well underway; NASA estimates that a possible MARS flight will span over 1100 days (14). With the emergence of deep space flight exploration comes heightened unmitigated risk factors (15–17). With these missions, the need for protecting our flight crews increases. As we continue to look toward a new frontier of space exploration it is imperative we are aware of the risks on the human brain with regard to prolonged periods of microgravity exposure and body fluid shifts, as well as the isolation and radiation that our astronauts face, in addition to other stressors. Perhaps harder to see, many molecular changes will arise as well as missions have longer durations, given our observations on shorter missions (18–24). Moreover, space exploration greatly increases structural changes in the brain as a result of the heightened intracranial and intraocular pressure changes (25). To date, investigations from the International Space Station (ISS) and other missions have demonstrated the vast changes the human brain experiences throughout space flight and will be discussed in more detail below.

## Stressors during space flight

**Figure 1:**
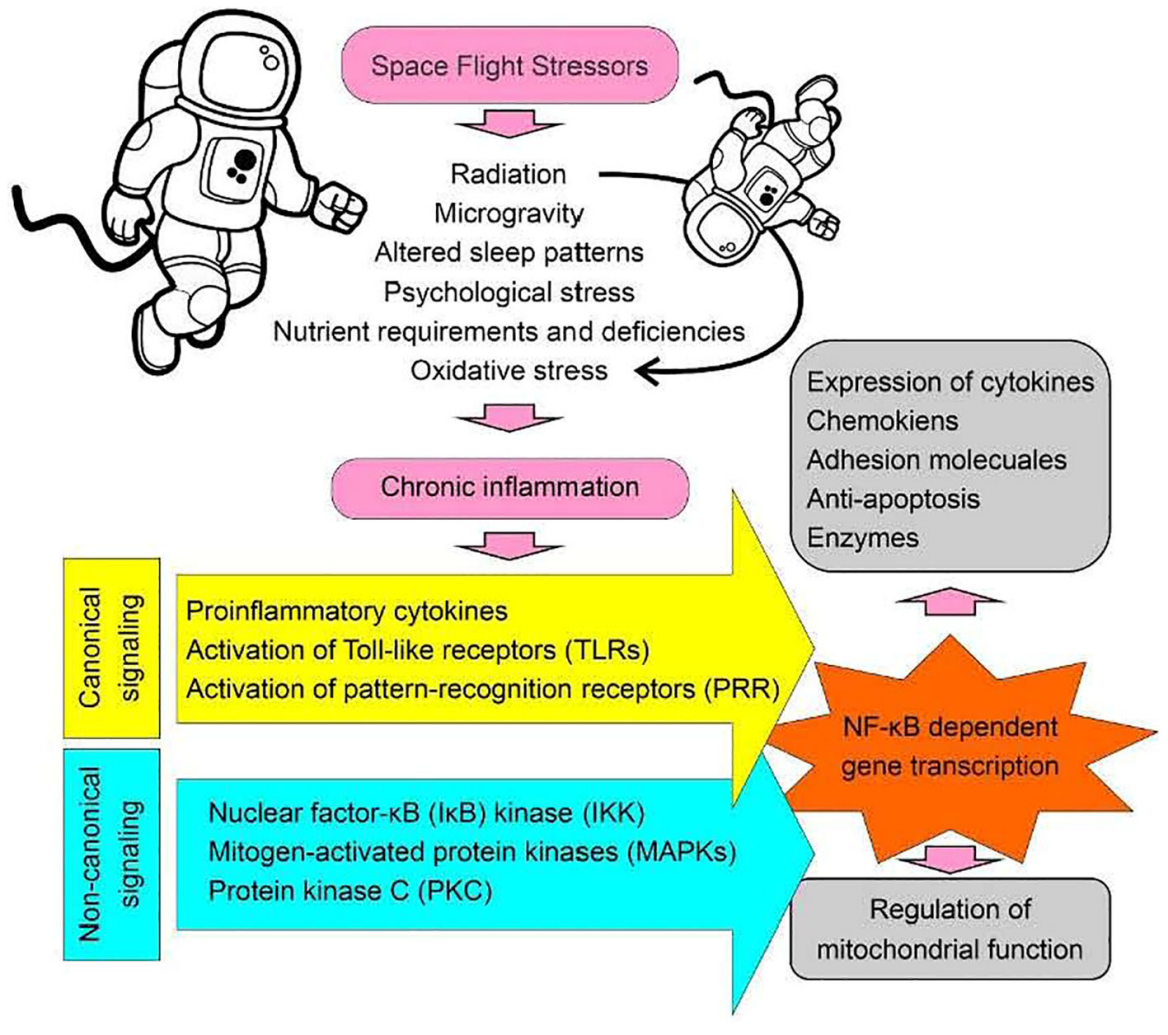
Space flight stressors. A diagrammatic presentation illustrating the factors associated with spaceflight that may contribute to the development of chronic inflammatory conditions, resulting in the activation of the NF-κB signaling pathway and subsequent effects on mitochondrial function. Canonical signaling refers to established pathways with common or standard features, such as the activation of pattern recognition receptors (PRRs). In contrast, non-canonical signaling involves the activation of modulators linked to an established pathway that does not fit into the canonical model. For example, mitogen-activated protein kinases (MAPKs) can act as common modulators for multiple signaling pathways converging through different receptor activations during inflammation. For instance, activation of the Purinergic Receptor P2X 7 (P2X7), which is an ATP-gated ion channel, leads to MAPK activation. Similarly, activation of the chemokine receptor C-X-C Motif Chemokine Receptor 4 (CXCR4) can also lead to MAPK activation through G-protein coupled receptor (GPCR) signaling. Thus, MAPK activation represents a non-canonical signaling event underlying inflammation”.

Space flight or living on a Moon or Martian colony is an extreme environment that forces us to think about survival, stress factors, and also creative countermeasures. Many of the stress factors are obvious (26–29), while others are yet to be determined (**Figure 1**). Countermeasures once developed may not only be protective for space flight crews, but also may find usefulness for Earth populations (30–33) and vice versa. Some of the changes seen with regard to cardiovascular efficiencies, bone and muscle loss, and vestibular system disturbances can be reduced with exercise and training, similar to programs for the elderly now used on Earth. However, long-term human space flight outside of the Moon’s orbit has not been studied exhaustively, and so additional and creative countermeasures will be needed. There are five main stressors the human body encounters during space flight: these are space radiation, microgravity, isolation and confinement, hostile or closed environments, and the distance from earth (24, 25). Radiation is energy that comes in the form of electromagnetic waves and particles. Sources of radiation are the Sun and cosmic microwave background (34, 35). Microgravity results in the near weightlessness humans experience in space, but humans still experience gravitational forces in space (36) given they possess mass and are subjected to a small gravitational force. These forces come from other celestial bodies and man-made objects. Lastly, space-faring humans experience social isolation and confinement inside spacecraft or on any space station (27, 37). For example, humans onboard spacecraft do not see anyone outside of their group for months at a time, except in video chats. Data suggest this creates a significant psychological impact that has yet to be fully appreciated in longer term missions.

### Space radiation

Radiation is a hazard to human health as it damages biological structures, if not shielded. The Sun is one of the biggest sources of radiation for humans during space flight (38–40). It emits all wavelengths in the electromagnetic spectrum, mostly in the infrared to ultraviolet range (2500 nm to 250 nm). At times, the Sun experiences solar particle events (SPEs) where protons at different energies are accelerated through interplanetary space (41). This event, specifically called coronal mass ejection, releases X-rays and gamma rays along with high-speed protons (42, 43). Moreover, galactic cosmic rays (GCRs) are a highly energetic background source of energetic particles that constantly bombard Earth (44). GCRs originate outside the solar system and are likely formed by explosive events such as supernova. GCRs consist of almost every element ranging from hydrogen to uranium, accompanied by sporadic heavier ions termed high-energy, high-charged particles (HZE). To model GCRs on earth, a GCR simulator (GCRsim) at the NASA Space Radiation Laboratory (NSRL) was developed (45).

Additionally, electrons and positrons are also present within this cosmic radiation spectrum (46). SPEs and GCRs are harmful and destructive to the human body without proper shielding (47, 48). However, proper shielding on space craft is currently impossible. In fact, current spacecraft hulls cause secondary scatter thus complicating the problem. Onboard the ISS, astronauts experience an average dose of 100 – 200 millisievert (mSv) per year. However, this dose would increase to 350 mSv per year for astronauts on a 3-year Mars mission (24). Simulated GCR exposure impacts cognitive and behavioral functions, synaptic integrity, and microglial activation (49). DNA double strand breaks also occur with radiation exposure as does overall telomere length shortening after space flight as compared to before space flight (50–52). However, during space flight telomeres elongate in peripheral blood, but shorten upon return to Earth and approach baseline levels during postflight recovery (53). Studies in mice have also shown that space radiation exposure can cause memory and learning impairments (46). However, the effects of radiation are often difficult to tease apart from the additional effects of microgravity.

### Microgravity

Another stressor that humans experience during long-term space flight is microgravity. Microgravity is the condition in which humans appear to be weightless (54). Onboard the ISS, astronauts experience microgravitational effects as they orbit around the Earth once every 92 minutes. As a result, in space humans experience weakened bone structure. It is estimated that accelerated bone loss occurs at a whole-body rate of 0.5-1% per month, due to the low gravitational forces experienced on the human body (55). Moreover, humans in space may experience vision problems, referred to as Space Associated Neuro-Ocular Syndrome (SANS), where the reasons for this are unclear, but some think it is caused by a brain upward shift (BUS) (56) due to a combination of microgravity and space radiation. Another idea has recently emerged (57), that points to mitochondrial dysfunction in SANS. The increase in the fluid present in the cranial region causes brain edema (58), and learning and neuroplasticity changes are some of the symptoms astronauts may experience (58, 59). Another study shows that the effects of microgravity include the crowding of brain tissue at the vertex and the expansion of the ventricular system due to the upward shift of the brain (60, 61). Overall, there exists a strong similarity between aging and microgravitational changes (62–66). Given this, some gerontologists use microgravity as a model of aging (66) or accelerated aging.

### Isolation and confinement

Confinement is a stressor that affects human mental health. Astronauts on board any space capsule or the ISS are confined to small quarters for prolonged periods of time. In addition to the effects on the hippocampus - a key brain structure playing a role in learning and memory, we must also examine the emotional distress that astronauts face as a result of sustained isolation periods. Emotional regulation by using emotion training has been studied to be a potential countermeasure during long term space flight (67). For example, when we look at the termination of the Soyuz T14-Salyut 7 mission in 1985, we can see that the main reason for its demise was linked to the crew’s depression and continuing decline in mental health (67). In periods of isolation, history has shown diminished cognitive resilience, passion, as well as the increase of anxiolytic based symptoms. The emotional changes experienced by astronauts may not only compromise their own well-being, but the safety of the other crew members and the flight in general. In addition to human studies performed, animal studies have shown that social isolation, immobilization, and changes in gravity can have dramatic effects on brain plasticity and spatial navigation (14). These studies have also shown how the stress of isolation actually disrupts hippocampal neurogenesis as well as impair hippocampal long-term potentiation (LTP) (67, 68), an experimental paradigm associated with synaptic plasticity and memory encoding.

### Hostile or closed environments

Research in environments such as Antarctica has served as a model for space missions (69). Hostile earth-based terrestrial environments are very useful as a testbed for future Mars colonies or other icy planets. These test environments have already given us some insight into challenges such as low water availability, high radiation levels, strong winds, rough terrain, and a geochemical habitat that might resemble other celestial bodies (70). Also essential in these environments is the study of human factors such as long-term interactions among an international crew. Other learnings can be gleaned from testing advanced life support equipment and conducting basic scientific research similar to the research that will be conducted on the Moon, Mars, or other planets. Some physiological changes that occur in Antarctica that might have relevance for space travel include circadian disruptions, immunosuppression, cardiometabolic alterations, changes in the quality of sleep, metabolic and neuroendocrine functioning, memory impairments, and psychological stress, to name a few (71). These changes can thus affect physical and cognitive performance. Also, the forced coexistence of diverse people from various countries are additionally faced with a range of cultural, political, linguistic, religious, and gender differences, that can also contribute to interpersonal problems and psychological stress. Countermeasures in the Antarctic environment to date have included dietary supplemental nutrition, meal scheduling, internet connectivity, and training in coping strategies and remote monitoring aids. Moreover, closed environments offer additional health issues. For example, space capsules and the ISS are closed environments that harbor various microbial communities (72, 73). Given this, challenges arise that include latent virus reactivation, microbial drug resistance, and changes in the gut microbiome (74). All of this happening at a time when dysregulation of the immune system occurs in space (75).

### Distance from Earth

The increased distance from Earth would also likely affect the mental health of humans on board a space craft, however, this aspect has not yet been directly tested. For example, Mars is on average, 140 million miles from Earth (but this distance ranges from 34 to 250 million miles) whereas the Moon is approximately 238,900 miles away. In other words, the Moon takes about 3 days to travel to where a trip to Mars could take 210-270 days or 7-9 months one way – roughly 145 times farther. Some estimate it might take 21 months to go to Mars and back. So, it is not surprising that data from past space flight missions suggest that spatial cognition and neural networks could be impaired during long duration space travel (14).

## Immune system function, inflammation, and NF-κB signaling

### Immune systems

The human immune system has two components: that is the innate immune system and the adaptive immune system (76). The innate system is the body’s rapid, but non-specific response system, while the adaptive system is specific and activated when the body is exposed to microbes or other foreign agents. The cells of the innate immune system include neutrophils, monocytes, natural killer (NK) cells, and proteins known as the complement proteins. However, the adaptive immune system utilizes T cells and B cells that need to be trained resulting in immunological memory. The systems are connected and work together during an immune response.

Data collected from a study from a long duration flight of over 140 days analyzed immune function before, during, and after the travel of twelve participants. While stress markers of cortisol in saliva went unchanged, investigators did observe an approximate 50% increase in monocytes and B cells as well as a 60% decrease in NK cells. Additionally, upon landing glutathione levels were found to be constant, but with an increased shedding of the cell-adhesion molecule, L-selectin or CD62L (77, 78).

Additional data thus far shows that dysregulated immunity is a primary challenge of space flight. Previous research focusing on persistent reactivation of the latent herpesvirus family demonstrates how this reactivation may act as a major indicator of reduced immunity and dysfunction of lymphocytes (78). A healthy immune system would be able to fight off the recurrent virus, however, due to the suppression of specific T lymphocytes, this evidence suggests there is a mechanism preventing macrophage functionality. Additionally, it has been reported that under conditions of microgravity, macrophages do not function as they normally would (79). In addition to the lessened cytokine production that has been seen, there has also been an observed dysfunctionality in the number of macrophages, leukocyte antigens, and oxidative burst reactions (79). The decrease in both the number of cells as well as an abnormality in their functionality will alter both the proinflammatory and anti-inflammatory mechanisms of the cell and organism; ultimately interfering with the immune response of the macrophages.

It is interesting to note data on differences between adaptive immunity and innate immunity. Adaptive immunity is our immune system response to a foreign substance or microorganism, such as after an infection or vaccination, whereas innate immunity is present at birth and lasts a person’s entire life. Through analysis we see that while aspects of adaptive immunity become dysfunctional, there is an enhancement of innate immunity (78). However, there is also an abnormality on the interaction of these two systems further exposing the crew members to potential altered disease states (78). Although the precise nature of immune deregulation must be further analyzed, numerous studies have shown that there remains an increased risk of allergic episodes, skin irritation, and recurrent virus infection; all pointing toward lessened immunity during space flight (80– 81). Additional in-flight and post-flight testing must be done in order to efficiently analyze the direct immune system abnormalities as well as advance alleviation strategies for decreased immunity in our astronauts for future long-term space flight (82).

### Inflammation

Inflammation is the natural response to injury and/or infection. It’s a defense mechanism that promotes healing an injury or fighting an infection. While acute inflammation is typically good, prolonged or chronic inflammation is not. Studies to date have shown radiation-induced neuroinflammation and changes in inflammatory cells after space radiation exposure (83). Past data has also shown the effect that cosmic radiation has on cognitive impairment via neuroinflammatory mechanisms. For example, HZE ions are the high energy component of GCRs that possess an electric charge higher than three. Past studies have implicated these ions as a major player in cognitive decline and abnormality, specifically in younger astronauts who still have developing neurocognitive systems and therefore may be more likely to experience cognitive issues (84). HZE has been noted to increase neuroinflammation due to mechanisms involving astrocyte activation (84). It is this incitement of both astrocytes and microglia that affect motor skills as a function of cognitive reduction. In order to better understand cognitive decline as it relates to inflammation we must further analyze the role that neuroinflammation plays in degeneration. One study, Cherry et al (85), correlated HZE particles with the development of Alzheimer’s Disease (AD) in mouse models that had previously shown a genetic predisposition for AD. Interestingly, the progression of the disease is supported by neuroinflammatory mechanisms through cellular adhesion, especially via Intercellular Adhesion Molecule 1 (ICAM-1) (85). Data from this study found that these highly positively charged particles act to increase ICAM-1 thus initiating the endothelial cells of the blood brain barrier; ultimately causing reduced clearance of amyloid plaques; a key hallmark of AD (85). In addition to memory impairment, activation of ICAM-1 has also been linked to mood disorders, especially bipolar disorder (84).

In a recent study, ten-week old male C57BL/6 mice were launched to the ISS using Space-X 12 for a 35-day mission (86). A digital counting technology (NanoStringTM) was used to evaluate gene expression profiles in the space flight mouse brain. The study results indicated that neuroinflammation and altered immune responses may be closely associated with space flight-induced stress and have an impact on the neuronal function that may result in chronic neuroinflammation and late neurodegeneration. More work is thus needed to identify key inflammatory components.

### Nuclear factor kappa B

NF-κB is a primary mediator of inflammation and immunity. Previous studies have shown that alterations in NF-κB signaling, are largely associated with disease states (87–89).

For example, NF-κB is found to play a significant role in changes in osteoblast activity, muscle atrophy, and immune dysfunction (90). In addition to the effects of NF-κB downregulation on the immune system, there is also evidence for similar effects on the musculoskeletal system. Previous research gives insight to NF-κB activation in musculoskeletal disorders including muscular atrophy, sarcopenia, and muscular dystrophy (91). Research assessing both sarcopenia and Duchenne muscular dystrophy have shown the upregulation of cytokine groups in the NF-κB cytokine pathway, suggesting that where there is muscle dystrophy, we will find an upregulation of NF-κB. NF-κB plays an undeniable critical role in neuronal cells when faced with neurotoxins as evidenced by elevated NF-κB activity in neurodegenerative disorders (92) and also in studies of cancer (93–95).

**Figure 2:**
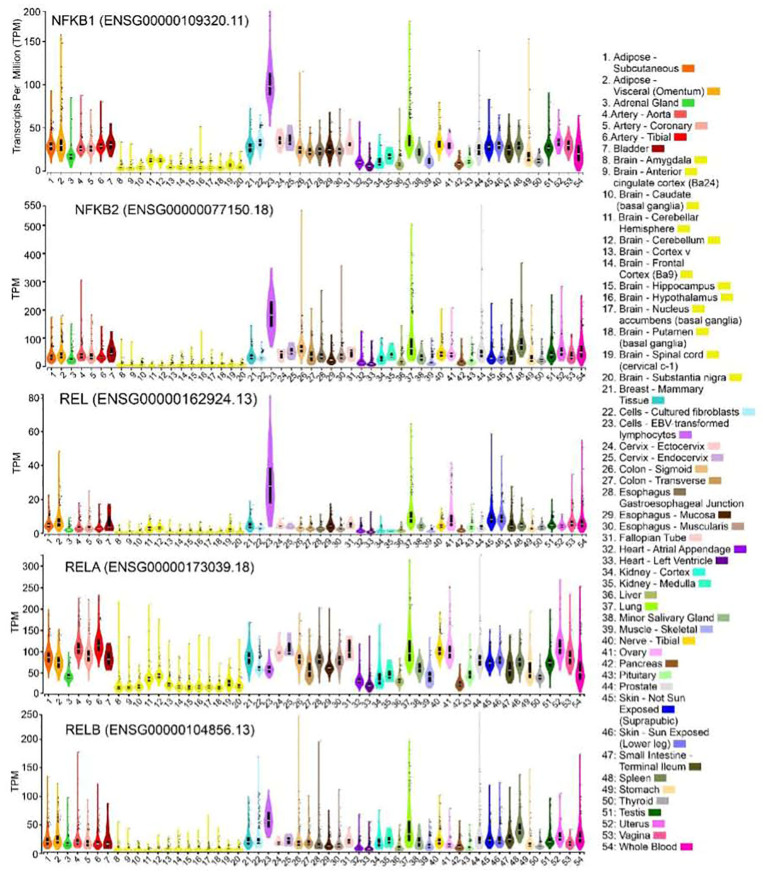
Expression of NF-κB family genes in primary human tissues. Violin plots showing expression for NFκB1, NFκB2, REL, RELA and RELB genes in 54 bulk human tissues derived from GTEx database. The expression values are shown in TPM (transcripts per million) calculated from a gene model with isoforms collapse to a single gene. The solid black colored boxplot plots showing the 25th percentile, the median (the 50th percentile, white horizontal bar), the 75th percentile, and outlying or extreme values (shown as solid black circles). Sample sizes are: subcutaneous adipose: 663; Visceral adipose: 541; Adrenal gland: 258; Aorta:432; coronary artery: 240; Tibial artery: 663; Bladder: 21; amygdala: 152; Anterior cingulate cortex (Ba24): 176; Caudate (basal ganglia):246; Cerebellar hemisphere: 215; Cerebellum: 241; Cortex: 255; Frontal Cortex (Ba9): 209; Hippocampus: 197; Hypothalamus: 202; Nucleus acumens (basal ganglia): 246; Putamen (basal ganglia): 205; Spinal cord (cervical c-1): 159; Substantia nigra: 139; Mammary Tissue: 459; Cultured fibroblasts: 504; EBV-transformed lymphocytes: 174; Cervix - Ectocervix: 9; Cervix - Endocervix: 10; Colon - Sigmoid: 373; Colon - Transverse: 406; Esophagus Gastroesophageal Junction:375; Esophagus - Mucosa: 555; Esophagus - Muscularis: 515; Fallopian Tube: 9; Heart - Atrial Appendage: 429; Heart - Left Ventricle: 432; Kidney - Cortex: 85; Kidney - Medulla: 4; Liver: 326; Lung: 578; Minor Salivary Gland: 162; Muscle - Skeletal: 803; Nerve - Tibial: 669; Ovary: 680; Pancreas: 328; Pituitary:283; Prostate: 245; Skin - Not Sun exposed (Suprapubic):604; Skin - Sun Exposed (Lower leg): 701; Small Intestine - Terminal Ileum: 187; Spleen: 246; Stomach: 359; Thyroid: 361; Testis: 653; Uterus: 142; Vagina: 156; Whole Blood: 755. The GTEx Project was supported by the Common Fund of the Office of the Director of the National Institutes of Health, and by NCI, NHGRI, NHLBI, NIDA, NIMH, and NINDS. The data used for the analyses described in this manuscript were obtained from the GTEx Portal (https://gtexportal.org/home/) on 06/24/2023 using GTEx Analysis Release V8 (dbGaP Accession phs000424.v8.p2).

The NF-κB family of transcription factors also exhibits widespread expression across various human tissues, as evidenced by RNA sequencing data available in the Genotype-Tissue Expression (GTEx) database (**Figure 2**). Notably, the genes *NF-κB1*, *NF-κB2*, *Rel*, and *RelB* show the highest levels of expression in EBV-transformed lymphocytes (median transcripts per million values: 98/181/27/53, respectively), surpassing other tissues (the range of median TPMs <50/100/10/36, respectively). In contrast, RelA expression demonstrated relatively higher levels in most tissues (TMPs range: 113-44) compared to the brain (TMPs: 42-14) and select other tissues (heart, pancreas, liver, kidney, testis, adrenal, and muscle TPMs: 42-19). The comparatively higher expression of NF-κB1 in human lymphocytes aligns with findings of defective immune function in mice with NF-κB1 deletions (1). In mice, NF-κB1 deletion has been associated with inflammatory arthritis (2), accelerated aging, and degenerative changes in the cortex and hippocampus (3), as well as alterations in sleep regulation following immune challenges (4). Furthermore, deletion of NF-κB2 in mice led to defective T-cell response and abnormal spleen and lymph node architecture, which is consistent with the observed high expression of NF-κB2 in human lymphocytes (5, 6). Similarly, loss of c-Rel in mice impaired T and B cell activation and caused resistance to systemic collagen-induced arthritis (2, 7, 8), while deletion of RelB in mice resulted in defects in antigen-presenting cell function (9), T-cell infiltration of organs (10), and skin inflammation (11). These immune-associated phenotypes align with the high-level expression observed in human lymphocytes for both Rel and RelB. On the other hand, RelA deletion in mice resulted in embryonic lethality (12), further supporting its relatively high-level expression in a large number of human tissues. This indicates that RelA is an important gene for the functioning of the majority of organs. It is conceivable that loss of this gene may lead to multiple organ failure, ultimately resulting in embryonic death.

### NF-κB in space flight studies

Prolonged space flight induces stress factors that can have long term consequences on the NF-κB gene family, possibly leading to immune dysregulation in astronauts. NF-κB signaling, central to many health conditions, is modulated across various cell types in both real or simulated space conditions (96, 97). Specifically, studies have shown that microgravity can impact T cell function, as seen in the “Soyuz 13S” mission where over 40 genes, including c-Rel, were downregulated (98). Additionally, NF-κB plays a role in mitochondrial interactions and functions. Bottero et al.’s study on Jurkat cells and the TNFα stimulation’s impact on mitochondrial fusion in cardiac myocytes exemplify this interaction (99, 100). NF-κB’s involvement is critical in regulating mitochondrial respiration—a key factor in brain metabolism and neurodegenerative conditions (101).

Given the centrality of NF-κB in both innate immunity and inflammation, understanding its modulation in space is essential. Targeted interventions that stabilize NF-κB activity may offer promising strategies to mitigate the adverse effects of space flight on the immune system, ensuring better health outcomes for astronauts during long-duration missions.

The NF-κB signaling pathway plays a crucial role in regulating innate immunity, which is significantly impacted by space flight conditions. Microgravity, a primary stressor during space missions, has been shown to disrupt NF-κB activation and its downstream effects on immune responses. Under normal conditions, NF-κB remains in the cytoplasm until stimulated by microbial-associated molecular patterns (MAMPs) or reactive oxygen species (ROS), leading to its translocation into the nucleus where it activates immune-related genes (102). However, during space flight, the combined effects of microgravity and cosmic radiation can profoundly disrupt NF-κB activation, leading to significant alterations in immune function. For instance, studies have demonstrated that exposure to space flight conditions, particularly microgravity, impairs NF-κB translocation in immune cells, resulting in reduced activation of T cells and suppression of cytokine production (62, 102, 103). This suppression is likely due to the altered mechanotransduction signals and impaired signal transduction pathways, such as those involving MyD88, a key adaptor protein in NF-κB signaling (102). Additionally, space flight-induced stress responses, such as elevated levels of corticosterone, can further modulate NF-κB signaling, compounding the immune dysfunction observed during space missions (102). These disruptions are also exacerbated by increased DNA damage and oxidative stress, leading to increased NF-κB activation and a dysregulated immune response (103). These findings underscore the critical need for targeted interventions to stabilize NF-κB activity, thereby mitigating the adverse effects of space flight on the immune system and enhancing astronauts’ health during long-duration missions (102–104).

Mitochondrial dysfunction is linked to aging and a plethora of neurodegenerative diseases due to their role in metabolic homeostasis and their unique replication mechanisms, making them particularly vulnerable during space flight (105–110). Multi-omic analyses indicate mitochondrial stress lies at the heart of the body’s systemic adjustments in space conditions (111). Experiments have demonstrated that microgravity disrupts the structural calibration of the cytoskeleton, altering mitochondrial distribution and potentially leading to deregulations in energy pathways like glycolysis and TCA cycles, and increasing reactive oxidative species (ROS) (112–115). High-level oxidative stress has been correlated with increased space flight duration, affecting ocular tissues and potentially cerebral arteries, suggesting the involvement of oxidative stress in space flight-induced health risks (116, 117).

The tissue-specific expression of NF-κB genes shows a notable prevalence in lymphocytes, with elevated expression levels of NFκB1, NFκB2, REL, and RELB potentially correlating to immune function dysregulation, characteristic of chronic inflammatory and neurodegenerative diseases—conditions possibly exacerbated by the stress of space flight (118–128). The widespread expression of RELA across tissues, its importance underscored by embryonic fatality upon deletion in mice, suggests it is crucial for the functioning of most organs (129). While multiple studies have highlighted the impacts of space conditions on mitochondria and NF-κB genes, the complex mechanisms remain to be fully elucidated, necessitating further research and experimentation in these areas to understand the implications of space travel on human health.

### Sex Differences in Brain Activity and Immunity

Research indicates that neurological and sensory processing differences between males and females exist, including variations in amygdala activity, vision sensitivity, and neuronal cell death. These differences may manifest in space exploration, such as the higher incidence of space sickness and post-flight instability observed more frequently in female astronauts, though data is limited due to gender imbalances in astronaut recruitment (130, 131).

Recent insights highlight significant sex-specific disparities, such as the higher occurrence of immediate post-flight orthostatic intolerance among female astronauts, potentially linked to lower vascular resistance in leg vessels (132, 133). This condition poses safety risks, necessitating further research and targeted countermeasures.

Females generally exhibit more robust immune responses than males, potentially providing better protection against infections during space missions. However, this heightened immune vigilance also correlates with a greater tendency toward autoimmune conditions, which requires more comprehensive health monitoring for female astronauts (132, 134–137).

Additionally, women are more susceptible to radiation-induced cancers, such as lung, thyroid, breast, and ovarian cancers, which may limit their time in space. This vulnerability, combined with generally longer life expectancies, underscores the need for careful mission planning to minimize long-term health risks (132; 138, 139).

Reproductive health concerns also arise, with both males and females potentially experiencing infertility from short-term exposure to ionizing radiation. Changes in the hypothalamic-pituitary-gonadal and hypothalamic-pituitary-adrenal axes have been observed during space travel, potentially affecting hormone levels and stress responses (140–144).

## Pharmacological, nutritional, and biological assessments and interventions

### Monitoring

Equipment and sensors for brain monitoring and assessing physiological brain parameters (and other organ systems) have been used routinely for studying the effects of space flight. This approach will be even more important for deep space travel and will be needed in conjunction with various interventions. To this end, longer-duration missions will need to bring along electroencephalogram (EEG) instrumentation, spectroscopic imaging systems, and ultrasound equipment (25). Larger instruments such as MRI, will not be feasible due to size and weight. However, a variety of changes are expected during space flight that include structural brain changes, alterations in the distribution of cerebrospinal fluid (CSF), and changes in cognitive performance. Other changes that will need monitoring include blood biomarkers (145), pharmacotherapy (146), nutritional status, and inflammatory changes.

### Drug use during space flight

**Figure 3:**
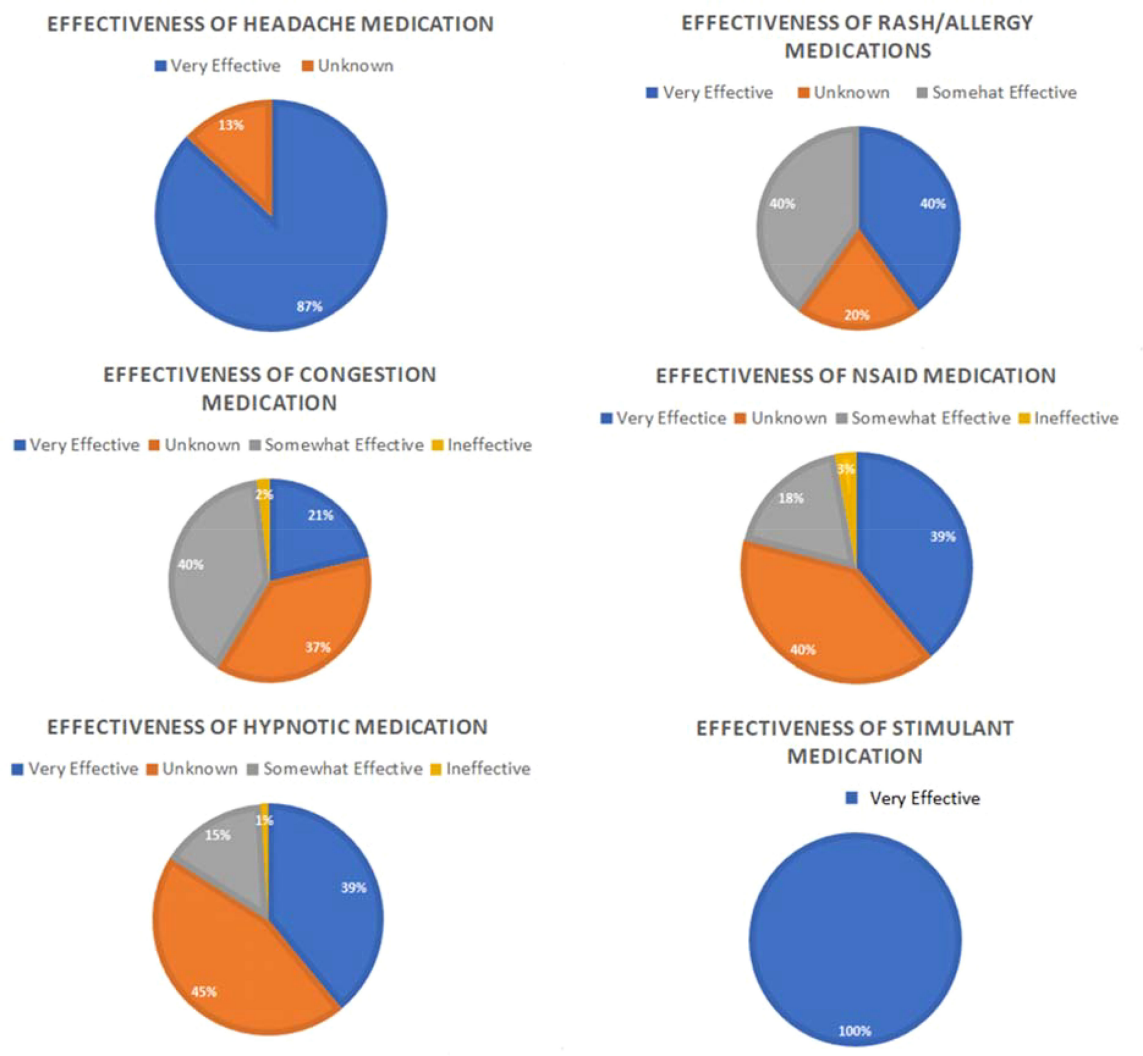
Medication effectiveness. Figure shows subjective reports of medication effectiveness for six clinical indications; stimulant, headache, rash/allergy, NSAID, hypnotic and congestion during ISS Expeditions 21-40%. It can be noted that medication reporting is voluntary hence it is not done by everyone who takes medication during the space flight. Modified from (147).

Medication access in space has been a topic of interest since the first human space flight. Although space medicine is classified as a broad clinical discipline, humans engaged in space flight and other aerospace activities are still facing challenges (146). During space flight, crew members normally take medications (**Figure 3**) for sleep, pain, skin conditions, motion sickness, illnesses, injuries, behavioral health problems, as well as space motion sickness based on consultation with their flight surgeon (146).

All medications available on board for the ISS crew are drugs that have been approved for over-the-counter or prescription usage by the Food and Drug Administration as shown in **Table 1** (148). Although the aerospace medical community relies heavily on the usage of corticosteroids, antibiotics, antivirals, antiemetics, antihistamines, cytokines, decongestants, adrenergics, mast cell stabilizers, anti-inflammatory agents, laxatives, antifungals, antidepressants, hypnotics, anesthetics, antihypertensives, and opioids, there are still concerns about the effectiveness of these medications during space flight (146). This is due to the fact that there has been a diminished response reported by crew members to the initial dose of some medications such as zolpidem when taken during flight (146).

**Table 1:** Medications used during space flight.

Acetaminophen	Loratadine
Acyclovir	Loperamide
Amoxicillin/Clavulanate	Melatonin
Aspirin	Metoprolol
Atorvastatin	Metronidazole
Azithromycin	Modafinil
Cefadroxil	Mupirocin
Centrum Silver Multivitamin	Nasal Cobolamine
Ciprofloxacin	Phenytoin
Clotrimazole	Progestin/Estrogen
Dextroamphetamine	Promethazine
Epinephrine	Pseudoephedrine
Fluconazole	Risedronate
Furosemide	Sertraline
Ibuprofen	Silver Sulfadiazine
Imipenem/Cilastin	Sulfamethoxazole/Trimethoprim
Levofloxacin	Temazepam
Levothyroxine	Triamcinolone
Lidocaine	Valacyclovir
	Vitamin D Supplement
	Women’s Once-A-Day Multivitamin
	Zolpidem

The table shows both over-the-counter and prescription drugs currently used on the ISS. Used with permission from Wotring et al. (148).

### Drug stability during space flight

During space flight, drug stability is one of the main concerns. A drug can be classified as unstable if there are any alterations in the chemical properties; for example, drug potency, dissolution and solubility or physical properties such as; changes in appearance and consistency (147). In order to maintain drug stability during space flight, it is important to take into consideration the shelf life of the medication as shown in **Table 2** (147). However, due to limited data on the stability of medications that have been in space, the ISS has given 87% of these medications a shelf life of less than 24 months (**Table 3**). Although, the electronic Medicines Compendium (eMC) and the Federal Shelf Life Extension Program (SLEP) has provided evidence that some medications can be given a shelf life of beyond 24 months (147). Medications that have been opened during space flight or repackaged are known to have altered drug stability due to degradation from temperature, humidity, and radiation (147). The prolonged impact of low-dose radiation on a medication during space flight is however not well understood (147).

**Table 2:** ISS formulary drugs and drug stability on Earth.

Drug	Dosage form	Shelf Life (mo)	Drug	Dosage form	Shelf Life (mo)
**Acetaminophen**	Tablet	36	**Loratadine**	Tablet	36
Acetazolamide	Tablet	48	Medroxyprogesterone	Tablet	60
Amoxicillin	Capsule	36-48	**Melatonin**	Tablet	36
Aspirin	Tablet	36	Metronidazole	Tablet	36
Atropine	Injectable	36	*Modafinil	Tablet	36
**Azithromycin**	Tablet	48-60	Mometasone	Nasal Spray	36
Bisacodyl	Tablet	36	Naloxone	Injectable	36
Clindamycin	Capsule	36	Olopatadine	Ophthalmic solution	36
Clotrimazole	Cream	36	Omeprazole	Capsule	36
Diazepam	Injectable	36	Ondansetron	Tablet	36
Diphenhydramine	Tablet/Injectable	36	Oxymetazoline	Nasal Spray	36
Doxycycline	Capsule	36-60	Promethazine	Tablet/Injectable	36
**Fluconazole**	Tablet	60	Pseudoephedrine	Tablet	36
Hydrocortisone	Cream	60	**Sertraline**	Tablet	60
**Ibuprofen**	Tablet	36	Sodium Chloride (Normal Saline)	Injection	36
Ketamine	Injectable	60	**Sulfamethoxazole/Trimethoprim**	Tablet	60
**Levofloxacin**	Tablet	36-60	Tamsulosin	Capsule	48
**Lidocaine**	Injectable	36	Triamcinolone	Cream	36
Lisinopril	Tablet	48	Valacyclovir	Tablet	36
*Loperamide	Capsule	60	*Zolpidem	Tablet	36

Medications that were found to be stable for extended shelf life as indicated. All drugs within this table are presented in the ISS formulary. Drugs in bold were found to be unstable on return from space flight and drugs marked with an asterisk (*) were found to have degradant products whose significance is unknown in post-space flight analysis. Used with permission from Blue et al. (147).

**Table 3:** ISS formulary drugs and drug stability beyond expiration on Earth.

Drug	Dosage form	Lots Tested	Mean Extension (mo.)	Extension range (mo.)
Amoxicillin	Tablet *	21	23	21-23
Bupivacaine	Injectable solution	3	88	79-95
Ceftriaxone	Injectable powder	4	60	44-69
**Ciprofloxacin**	Tablet	242	55	12-142
Cimetidine	Tablet	242	55	12-142
Dexamethasone	Injectable solution	7	61	24-93
Diphenhydramine	Injectable solution	12	76	33-126
Doxycycline	Capsule	13	50	37-66
Guaifenesin	ER Tablet	7	85	39-122
Ketamine	Injectable solution	6	64	42-87
Meperidine	Injectable solution	6	89	31-128
Morphine	Injectable solution	13	89	35-119
Naloxone	Injectable solution	10	77	60-95
**Phenytoin**	Injectable solution	5	63	29-100
**Promethazine**	Injectable solution	9	51	28-73

Medications extracted from Lyons et al. (149) that were found to be stable by indicated studies beyond package expiration dates in terrestrial conditions. All medications shown are on the ISS formulary. Drugs in bold were found to be unstable after space flight in one or more space flight stability studies in contrast to terrestrial study results. Used with permission from Blue et al. (147).

### Medication Use for Emergencies

Medications used for emergency purposes during space flight should be selected based on their usefulness and effectiveness in treating a wide range of indications (150). Medications typically used in the case of emergency during space travel are; acetaminophen, ampicillin, atropine, dexamethasone, diazepam, diphenhydramine, donnatal, epinephrine, erythromycin, hydroxyzine, cephalexin, lidocaine, meperidine, morphine, nitroglycerin, penicillin, prochlorperazine, promethazine, and tetracycline (150). All of which are listed on the ISS formulary.

### Supplements during space flight

Nutrition plays a vital role in space travel. It is important that each individual’s nutritional and metabolic needs are being met in order to enhance and maintain normal requirements and their overall emotional well-being (151). Recommendations have been made that crew members must receive nutrition that provides reasonable support to prevent negative effects such as, immune deficiency, oxidative stress, and bone and muscle loss (151).

The development of space nutrition delivery systems from primarily aluminum tubes in the 1960s to rehydrated foods to now more palatable food options that can be refrigerated and reheated on the spacecraft, aims to mimic the daily requirements consumed by humans on Earth. The World Health Organization (WHO) recommends a macronutrient composition with an average of 55% carbohydrates, 30% lipids and 15% protein as the bare minimum (151).

Metabolic stress and long-term radiation were found to suppress the immune system, cause increases in the metabolic rate, and increase a crew member’s risk of cardiovascular issues (152). In order for these long-term effects associated with improper nutrition to be prevented, nutritional countermeasures for the effects of microgravity, reduction in salt intake, an increase in unsaturated fatty acids and a decrease in saturated fatty acids, increased calcium and Vitamin K intake has been put in place to prolong long term complications associated with poor nutrition (151).

The majority of supplements used during space flight is for improving nutrition. B vitamin status (riboflavin, folate, and vitamin B6) has been associated with ophthalmic abnormalities during and after flight. This is due to multiple factors including the one-carbon metabolic pathway. Crew members who were found to have ophthalmic changes on return from space had higher dehydroepiandrosterone (DHEA) and DHEA-sulfate before flight as well as a higher testosterone response while in flight (153). These crew members were also found to have higher concentration markers that reflected insulin resistance and altered carbohydrate metabolism (153). As a result, studies are still being done to prove whether B vitamin supplementation can prevent these ocular changes during and after space flight.

### Future biological procedures

For long-duration space flights, the possibility of blood transfusions is being discussed, particularly for crew members at risk for hemorrhage events and circulatory issues (154). These procedures are crucial not only for physical health but also for maintaining cognitive resilience. Studies have shown that long-duration space flight can be associated with fluctuating red blood cell counts and plasma concentrations (154). These hemodynamic changes, including shifts in fluid towards the head, can lead to decreased plasma volumes and diuresis, potentially compromising oxygen delivery to the brain and other vital organs. Inadequate oxygenation and nutrient supply to the brain can impair cognitive function, decision-making, and overall mental performance, which are critical for mission success. By providing fresh blood through a “floating” blood bank design, it would be possible to stabilize these physiological parameters, thereby supporting both physical health and cognitive resilience in astronauts. Additionally, other types of transfusions, such as mitochondrial and platelet transfusions, currently being explored for Earth-based applications, might also help maintain cellular and neurological health in space (155, 156). Furthermore, the development of biobanking for stem cells and rehydration of freeze-dried biologicals, both for Earth and space applications, could offer significant benefits, including potential therapies for neurodegenerative conditions that could arise during prolonged missions (157–159). Establishing biobanks on Moon or Martian colonies could provide a critical resource for maintaining the health and cognitive function of future space explorers.

## Conclusion and recommendations

The challenging environment of deep space poses significant physiological and psychological risks to astronauts, urging the need for the development of comprehensive countermeasures to protect their health during extended missions. This review highlights the critical importance of understanding and mitigating the effects of space flight on brain function, immune responses, and overall brain health. Key processes such as inflammation, mitochondrial dysfunction, and NF-κB signaling play central roles in the physiological changes observed during space travel, with potential long-term consequences that extend beyond the mission.

Space flight accelerates aging-like processes, including immune dysregulation, oxidative stress, and cognitive decline, underscoring the need for targeted interventions. These interventions should focus on maintaining mitochondrial health, regulating immune responses, and protecting against radiation-induced damage. Pharmacological, nutritional, and biological strategies will be essential in minimizing the risks associated with long-duration space missions.

Additionally, the sex-specific differences observed in response to space flight stressors must be carefully considered when developing countermeasures. Female astronauts, in particular, face unique challenges such as increased susceptibility to radiation-induced cancers and a higher incidence of post-flight orthostatic intolerance.

Looking ahead, further research is needed to explore the underlying mechanisms driving these physiological changes and to refine the countermeasures necessary to mitigate their impact. Studies involving larger species and long-term space flight simulations will be vital in translating these findings into effective strategies for human health protection in space. Moreover, the lessons learned from space medicine have the potential to inform our understanding of aging and disease on Earth, offering broader applications for human health.

As humanity prepares to embark on longer and more distant space missions, the continued advancement of space medicine will be paramount in safeguarding the well-being of our astronauts and ensuring the success of their missions. Collaborative efforts between space agencies, research institutions, and the medical community will be essential in overcoming the challenges of space exploration and unlocking the full potential of human space flight.
